# Emerging Therapeutic Strategies for Nrf2-Associated Skin Disorders: From Photoaging to Autoimmunity

**DOI:** 10.3390/antiox15010069

**Published:** 2026-01-06

**Authors:** Hyeong Jae Kim, Jeong Hee Hong

**Affiliations:** Department of Physiology, College of Medicine, Lee Gil Ya Cancer and Diabetes Institute, Gachon University, 155 Getbeolro, Yeonsu-gu, Incheon 21999, Republic of Korea; fgfgfg1125@gachon.ac.kr

**Keywords:** Nrf2, oxidative stress, photoaging, autoimmune diseases, fibrotic disease

## Abstract

Nuclear factor erythroid 2-related factor 2 (Nrf2) is a master regulator of the cellular antioxidant response, modulating redox homeostasis, detoxification, and cytoprotective pathways. Impaired Nrf2 signaling has been associated with a wide range of cutaneous pathologies, including photoaging, autoimmune disorders, and inflammation. In this review, we highlight roles of Nrf2 in the physiological and pathological mechanisms underlying oxidative stress, autoimmunity-associated skin damage, and fibrotic diseases, with a particular emphasis on photoaging, psoriasis, vitiligo, and autoimmune-associated skin fibrosis. Furthermore, we elucidate several natural bioactive compounds, their therapeutic applications, and delivery platforms for mitigating oxidative stress-mediated skin damage through the modulation of Nrf2 signaling, aiming to translate basic insights into clinical interventions for oxidative stress-driven skin disorders.

## 1. Oxidative Stress and Nrf2 in Skin

Nuclear factor erythroid 2-related factor 2 (Nrf2) is a ubiquitously expressed, redox-sensitive transcription factor that mediates cellular protection against oxidative stress, inflammation, and dysregulated immune responses [1,2]. Under homeostatic conditions, Nrf2 is sequestered by Kelch-like ECH-associated protein 1 (Keap1) and undergoes proteasomal degradation in the cytoplasm [3]. However, upon activation, Nrf2 translocates to the nucleus and binds to antioxidant response elements (AREs) in association with small Maf proteins to induce the expression of antioxidant genes, such as p62, heme oxygenase-1 (HO-1), NAD(P)H:quinone oxidoreductase 1 (NQO1), glutathione S-transferase (GST), and glutathione peroxidase (GPx) [4,5,6]. This mechanism preserves mitochondrial function and reduces reactive oxygen species (ROS) levels during oxidative stress [7,8,9,10,11].

Oxidative stress is recognized as a primary risk factor for various dermatosis [12]. It is characterized by an imbalance where the elevated production of ROS or reactive nitrogen species exceeds the capacity of antioxidant systems to neutralize these reactive molecules. ROS mediate damage to DNA, proteins, and lipids and activate inflammatory pathways that contribute to skin carcinogenesis and conditions such as psoriasis [13,14]. Patients with psoriasis exhibit increased ROS production and diminished antioxidant capacity [15], with disease severity and duration correlating with systemic oxidative stress levels [16]. Within psoriatic lesions, keratinocytes, activated neutrophils, and lymphocytes generate substantial amounts of ROS and free radicals, which subsequently exacerbate inflammation and keratinocyte hyperplasia [17,18]. Furthermore, excessive ROS levels activate the mitogen-activated protein kinase (MAPK)/activator protein-1 (AP-1) pathway [19], inducing matrix metalloproteinase (MMP) expression and subsequent collagen degradation. This activation also triggers the nuclear factor kappa-light chain enhancer of activated B cells (NF-κB) signaling pathway, which promotes the senescence-associated secretory phenotype (SASP) and the secretion of proinflammatory cytokines [20,21]. These processes drive cellular senescence, resulting in reduced skin elasticity and wrinkle formation [22]. In addition, ROS are one of the driving forces in the progression of skin fibrosis [23,24,25].

Therefore, understanding the role of Nrf2 in ROS-associated skin pathophysiology and its therapeutic implications may provide valuable insights for developing novel strategies against various skin disorders. In this review, we highlight the pivotal roles of Nrf2 in skin development and the pathological mechanisms underlying inflammatory and oxidative stress-related skin diseases, with a particular emphasis on photoaging, psoriasis, vitiligo, and skin fibrosis.

## 2. Methodology

This review comprises studies retrieved from PubMed and focuses on the roles of Nrf2 in various forms of skin damage and disease, with particular emphasis on photoaging, autoimmune disorders such as psoriasis and vitiligo, and autoimmune-associated skin fibrosis. Additionally, this review explores the involvement of Nrf2 in skin proliferation and differentiation, as well as its association with oxidative stress in the skin. Search terms included combinations of keywords, including Nrf2, skin, autoimmunity, oxidative stress, melanocytes, melanogenesis, proliferation, and differentiation. Studies focusing on efficacy evaluation without investigation of underlying molecular mechanisms were excluded.

## 3. The Role of Nrf2 in Skin Photoaging

Repeated and excessive ultraviolet B (UVB) exposure of skin cells generates substantial reactive oxygen species (ROS), triggering a state of cellular oxidative stress [26]. ROS stimulate various signaling pathways and initiate biological processes, including cell death, cellular senescence, and inflammation [27]. Specifically, UVB radiation mediates wrinkle formation, altered skin hydration, hyperkeratosis, collagen degradation, and inflammatory cell infiltration. Various bioactive natural compounds exert protective effects against oxidative stress-mediated skin damage through the modulation of Nrf2 signaling. We discuss several natural compounds to highlight their Nrf2-mediated oxidative stress defense properties in skin tissues and summarize their mechanisms in Table 1.

### 3.1. Salvianolic Acid B

Salvianolic acid B (Sal-B) is the most abundant and bioactive compound derived from *Salvia miltiorrhiza*, possessing potent anti-inflammatory and antioxidant properties [28]. UVB radiation-induced cellular senescence is protected by the Sal-B-mediated Nrf2 expression in human dermal fibroblasts (HDFs) [29]. UVB-induced skin wrinkle formation and epidermal thickening are inhibited through Nrf2 activation and the enhanced oxidative stress by UVB is attenuated through Nrf2 activation by recovering mitochondrial function and expression and activity of superoxide dismutase (SOD) in Sal-B-exposed HDFs [29]. Furthermore, UVB-mediated diminished dermal collagen and moisture content are reversed through Nrf2 activation by treatment with Sal-B in mouse dorsal skin [29]. In addition to skin tissue, Sal-B upregulates the Nrf2 antioxidant signaling pathway and exerts protective effects against oxidative damage triggered by subarachnoid hemorrhage [30].

### 3.2. Eisenia bicyclis Extract

*Eisenia bicyclis* (Kjellman) Setchell (*E. bicyclis*) is a common brown alga belonging to the family *Laminariaceae* [31]. Its extract, *Eisenia bicyclis* extract (EBE), has been shown to exhibit anti-inflammatory [31], anti-thrombotic [32], anti-diabetic [33], and neuroprotective effects [34]. UVB radiation-induced MMP-1 expression is attenuated through downregulation of the MAPK/AP-1 pathway and upregulated tissue inhibitor of metalloproteinases (TIMP) expression, such as TIMP1 and TIMP2, by treatment with EBE in HDFs [35]. Moreover, UVB radiation-induced collagen degradation and diminished skin moisture contents are reversed through activation of the mothers against decapentaplegic homolog family member (SMAD) pathway by treatment with EBE in HDFs and mouse skin tissues [35]. EBE treatment reduces UVB radiation-induced ROS production through Nrf2 activation, which enhances the expression of GPx1 and HO-1 in human keratinocytes [35]. In addition, wrinkle formation and epidermal thickening caused by UVB are reduced by oral administration of EBE in mouse dorsal skin [35].

### 3.3. Buds of Chrysanthemum morifolium

The buds of *Chrysanthemum morifolium* (*C*. *morifolium*) (BCM), which belongs to the *Asteraceae* family [36], possess antioxidant, anti-inflammatory, and analgesic properties [37]. Treatment with extract of BCM (EBCM) attenuates UVB radiation-induced MMP-1 expression through downregulation of the MAPK pathway in both human foreskin fibroblasts and human keratinocytes [38]. Elevated ROS production is reduced through upregulated Nrf2/NQO1/HO-1 signaling and glutathione (GSH) content by treatment with EBCM in UVB-irradiated human foreskin fibroblasts and human keratinocytes, thereby protecting skin water and collagen content, as well as maintaining epidermal thickness in UVB-exposed mouse skin [38].

### 3.4. Resveratrol

Resveratrol is a polyphenolic compound widely found in various plants, including the skin of grapes, *Veratrum nigrum* Linnaeus, and *Reynoutria japonica* Houttuyn [39]. Recent research has demonstrated that resveratrol possesses a wide range of pharmacological activities, including antioxidant [40], anti-inflammatory [41], and anti-apoptotic [42] effects. UVB radiation-mediated dysregulated skin pathology and inflammatory cell infiltration are alleviated by treatment with resveratrol in mouse skin tissues [43]. Treatment with resveratrol scavenges ROS production induced by UVB radiation through Nrf2/NQO1/HO-1 activation by enhancing expression of GSH, GPx, and SOD in human keratinocytes and mouse skin tissues [43]. Moreover, inflammation induced by UVB is inhibited through attenuated cyclooxygenase (COX)-2 and MAPK signaling by treatment with resveratrol in human keratinocytes and mouse skin tissues [43]. In addition, modified resveratrol application has been studied. Pterostilbene, a natural dimethoxy derivative of resveratrol, prevents UVB-induced skin wrinkling, erythema, and folding, while also exhibiting anti-carcinogenic effect through the activation of Nrf2-dependent oxidative stress defense systems [44].

### 3.5. Paeoniflorin

Paeoniflorin, a monoterpene glycoside isolated from the roots of *Paeonia lactiflora* [45], has been reported to exhibit various biological properties, including anti-inflammatory [46], anti-apoptotic [47], and antioxidant [48] activities. Treatment with paeoniflorin alleviates UVA-induced apoptosis in HDFs [49]. Moreover, treatment with paeoniflorin mitigates UVA-induced oxidative stress by upregulating the Nrf2/HO-1/NQO1 signaling pathway in HDFs [49]. Additionally, treatment with paeoniflorin in the presence of overexpressed lipid-binding protein perilipin 2 downregulates oxidative stress and promotes fibroblast proliferation in UVA-exposed HDFs [49].

### 3.6. Aloe vera

The genus *Aloe vera* has been known to possess various medicinal properties, including antioxidant, anti-inflammatory, anti-microbial and anti-carcinogenic effects, and is particularly relevant to the treatment of skin disorders and tissue repair [50,51]. Treatment with *Aloe vera* gel-derived nanoparticles (agDNPs) or *Aloe vera* rind-derived nanoparticles (arDNPs) mitigates UV-induced oxidative stress by suppressing Keap1 expression and activating Nrf2/NQO1/HO-1 signaling in human keratinocytes, HDFs, and mouse skin tissues [52]. Furthermore, treatment with agDNPs or arDNPs inhibits cellular senescence by upregulating the expression of collagen type I alpha 1 chain and nuclear laminar protein lamin B1 as well as induction of cell cycle arrest by attenuating p16, p53, and p21 expression in UV-exposed HDFs and mouse skin tissues [52]. Furthermore, UV-induced epidermal thickening, collagen depletion, and loss of elastic fibers are effectively restored by treatment with agDNPs or arDNPs in the dorsal skin [52], suggesting that an efficient delivery strategy using *Aloe vera* bioactive components exerts beneficial effects on photoaged skin.

### 3.7. Rosa davurica

*Rosa davurica*, a member of the Rosaceae family [53], exhibits antioxidant, anti-HIV, antiviral, antibiotic, and anti-atopic dermatitis activities [54,55]. UVB-induced ROS production is scavenged through promoted Nrf2/NQO1/HO-1 signaling by treatment with extract of *Rosa davurica* leaf (RDLE) in human keratinocytes [56]. Furthermore, treatment with RDLE attenuates the secretion of MMPs and interleukin (IL)-6 through downregulated MAPK/AP-1 signaling and enhances procollagen type 1 expression through TGF-β activation, thereby increasing anti-aging activity in UVB-exposed human keratinocytes [56].

### 3.8. Limonene

Limonene, a naturally occurring cyclic monoterpene found in citrus and conifer plants [57,58], possesses various biological activities, including antioxidant, antidiabetic, anticancer, and anti-inflammatory properties [57]. Treatment with limonene induces nuclear accumulation of Nrf2 and the consequent activation of HO-1/NQO1/γ-glutamate-cysteine ligase catalytic subunit signaling through activation of the c-Jun N-terminal kinase (JNK)/stress-activated protein kinase and phosphoinositide 3-kinase (PI3K)/protein kinase B (PKB) pathway, thereby scavenging ROS production in UV-exposed human keratinocytes [59]. In addition, treatment with limonene enhances skin barrier function by upregulating tight junction expression, including occludin and zonula occludens-1, in UV-exposed human keratinocytes [59].

### 3.9. Orientin

Orientin, a naturally occurring flavonoid found in several medicinal plants such as *Passiflora incarnata*, bamboo leaves, and *Ocimum sanctum*, exerts a wide range of biological activities, including antioxidant, anti-inflammatory, antiviral, and anti-bacterial effects [60,61,62,63,64]. Treatment with orientin reduces ROS levels by activating adenosine monophosphate-activated protein kinase (AMPK) through nuclear accumulation of Nrf2 and activation of the NQO1/HO-1 pathway as well as downregulated Bcl-2-associated X protein (Bax) expression and upregulated B-cell lymphoma 2 (Bcl-2) expression, thereby alleviating mitochondrial damage in UV-exposed human keratinocytes [65]. In addition, UV-induced inflammation is suppressed through attenuated cytokines expression and enhanced Nrf2 expression by treatment with orientin in human keratinocytes [65]. In parallel with the in vitro system, UV-induced dermal symptoms, such as epidermal thickness, erythema, and roughness, are alleviated through enhanced AMPK/Nrf2/HO-1/NQO1 signaling in the skin tissues of a photoaged mouse model [65].

**Table 1:** Mechanisms of bioactive compounds in skin photoaging and diverse effects.

Bioactive Compound	Related Mechanism for Alleviation of Photoaging	Effects	Models	Refs.
Sal-B	Nrf2 activation	Anti-inflammatory and antioxidant properties	UVB-radiated HDF	[28,29]
EBE	Downregulation of MAPK/AP-1 pathway, upregulated TIMPs expression, activation of SMAD, Nrf2 activation	Anti-inflammatory, anti-thrombotic, anti-diabetic, and neuroprotective effects	UVB-radiated HDF, human keratinocytes, and mouse skin	[31,32,33,34,35]
EBCM	Downregulation of MAPK pathway, upregulated Nrf2/NQO1/HO-1 signaling and GSH content	Anti-oxidant, anti-inflammatory, and analgesic effects	UVB-radiated human foreskin fibroblasts, human keratinocytes, and mouse skin	[37,38]
Resveratrol	Nrf2/NQO1/HO-1 activation, attenuated COX-2 and MAPK signaling	Antioxidant, anti-inflammatory, and anti-apoptotic effects	UVB-radiated human keratinocytes and mouse skin tissues	[40,41,42,43]
Paeoniflorin	Upregulation of Nrf2/HO-1/NQO1 signaling	Anti-inflammatory, anti-apoptotic, and antioxidant effects	UVA-radiated HDF	[46,47,48,49]
*Aloe vera*	Suppressed expression of Keap1 and activated Nrf2/NQO1/HO-1 signaling, upregulated expression of collagen type I alpha 1 chain and nuclear laminar protein lamin B1, and attenuated p16, p53, and p21 expression	Antioxidant, anti-inflammatory, anti-microbial, and anti-carcinogenic effects	UV-exposed HDF and mouse skin tissues	[50,51,52]
Limonene	Activated Nrf2/HO-1/NQO1/γ-glutamate-cysteine ligase catalytic subunit signaling and enhanced JNK/PI3K/PKB pathway	Antioxidant, anti-diabetic, anti-cancer, and anti-inflammatory effects	UV-exposed human keratinocytes	[57,59]
Orientin	Enhanced AMPK/Nrf2/HO-1/NQO1 signaling, downregulated Bax expression, and upregulated Bcl-2 expression	Antioxidant, anti-inflammatory, anti-viral, and anti-bacterial effects	UV-exposed human keratinocytes and photoaged mouse model	[60,61,62,63,64,65]

Abbreviations: Sal-B, Salvianolic acid B; Nrf2, nuclear factor erythroid 2-related factor 2; UVB, ultraviolet B; HDF, human dermal fibroblasts; EBE, *Eisenia bicyclis* extract; MAPK, mitogen-activated protein kinase; AP-1, activator protein-1; TIMPs, tissue inhibitor of metalloproteinases; SMAD, mothers against decapentaplegic homolog family member; EBCM, Extract of buds of *Chrysanthemum morifolium*; NQO1, NAD(P)H:quinone oxidoreductase 1; HO-1, heme oxygenase-1; GSH, glutathione; COX-2, cyclooxygenase-2; UVA, ultraviolet A; Keap1, Kelch-like ECH-associated protein 1; JNK, c-Jun N-terminal kinase; PI3K, phosphoinositide 3-kinase; PKB, protein kinase B; AMPK, adenosine monophosphate-activated protein kinase; Bax, Bcl-2-associated X protein; Bcl-2, B-cell lymphoma 2.

## 4. The Role of Nrf2 in Autoimmune Diseases

### 4.1. Psoriasis

#### 4.1.1. Nrf2-Associated Mechanism and Bioactive Compounds in Psoriasis Therapy

Psoriasis is an autoimmune skin disease characterized by keratinocyte hyperproliferation, immune cell infiltration, and persistent pruritus [66,67]. Nrf2 levels have been known to associate with psoriasis symptoms. For instance, *Arpc4*-null mice exhibit depletion of the Arp2/3 complex which is involved in the maintenance of keratinocyte shape and display psoriasis-like symptoms alongside enhanced expression of Nrf2 [68]. Moreover, psoriatic keratinocytes and human skin reveal downregulation of Arpc4 and Nrf2, both of which are associated with keratinocyte morphological regulation [68]. Furthermore, keratinocyte proliferation is promoted through the upregulated interaction of Nrf2 with keratin (K) 6, K16, and K17 in IL-17- or IL-22-stimulated human keratinocytes [69]. Application of imiquimod (IMQ) to murine dermal tissue has been used in psoriasis-mimic models. Nrf2 knockdown alleviates psoriatic symptoms, such as enhanced epidermis thickness, erythema, and hyperplasia, in an IMQ-induced psoriasis mouse model [69]. There are several studies to elucidate the roles of Nrf2 modulation-based bioactive compounds and these are summarized in Table 2.

• Gallic acid: Gallic acid is widely found in fruits and vegetables and possesses various biologic properties, including antioxidant and anti-inflammatory activities [70,71]. For instance, expressions of K16 and K17 are increased in the epidermis of psoriatic lesions [69,72,73]. Treatment with gallic acid attenuates psoriasis-like skin disease through the downregulation of K16 and K17 in both in vitro and in vivo models [74];
• Monomethyl fumarate: Monomethyl fumarate (MMF) is considered a potential candidate against psoriasis through enhanced expression of Nrf2 [75]. MMF treatment induces the protein expression of Nrf2 and Nrf2-associated oxidative stress responsive effectors in murine primary keratinocytes [75]. In addition, MMF treatment stimulates the expression of aquaporin 3 (AQP3), which is implicated in keratinocyte differentiation [75];
• Dimethyl fumarate: Dimethyl fumarate (DMF) has been used as anti-inflammatory, antiproliferative, and immunomodulatory effects [76,77,78]. Treatment with DMF induces apoptosis through increased p53 expression and decreased Bcl-2 expression, which is accompanied by elevated ROS production, and subsequently enhances the expression of Nrf2, HO-1, and steroid hormone metabolism-associated enzyme aldo-keto reductase family 1 member C3 (AKR1C3) in human keratinocytes [79]. In addition, treatment with DMF reduces inflammation and epidermal thickness, while increasing keratinocytes differentiation in an Nrf2-dependent manner in the IMQ-induced mouse model [80];
• Astilbin: Astilbin, a bioactive compound extracted from the medicinal herb *Smilacis glabrae*, has been reported to induce various effects, including anti-inflammatory [81], antioxidant [82], and antitumor [83] activities. Astilbin reduces ROS production through the nuclear translocation of Nrf2, thereby attenuating the proliferation of inflammatory cytokine-stimulated keratinocytes [84];
• Tussilagonone: Tussilagonone is an isolated compound from buds of *Tussilago farfara* and reveals Nrf2-associated anti-inflammatory properties [85]. This research group has also reported that tussilagonone attenuates psoriatic skin inflammation through the Nrf2/antioxidant response element (ARE) pathway in HaCaT keratinocytes and an IMQ-induced mouse model [86];
• Ambroxol: Ambroxol is isolated from *Adhatoda vasica* and is considered a multipurpose antioxidant, antifungal, antibacterial, antiviral, and antifibrotic compound [87,88]. Ambroxol also exhibits the anti-inflammatory effects in IMQ-induced skin tissues, characterized by the attenuation of the skin proliferative marker Ki67 [89];
• Mn-HyCO/Ru-HyCO: Manganese (Mn)/ruthenium (Ru) hybrid carbon monoxide (Mn-HyCO/Ru-HyCO), which consists of Mn- and Ru-based CO-releasing molecules (CO-RMs) conjugated to fumaric esters [90], enhances the Nrf2/HO-1 pathway and reduces epidermal thickness and scaling index in IMQ-induced mouse skin [90]. In addition, application of HyCOs induces anti-inflammatory effects and reduces skin wounds [90];
• Sulforaphane: Sulforaphane (SFN) is an organosulfur compound isolated from cruciferous vegetables and possesses antioxidant, anticancer, and anti-inflammatory effects [91,92,93]. Psoriasis symptoms, inflammation, and superoxide production are attenuated through Nrf2 activation by treatment with SFN in the IMQ-induced psoriasis mouse model or IL-22- or tumor necrosis factor (TNF)-α-stimulated human keratinocytes [94];
• Tryptanthrin: Tryptanthrin (TPT) is a natural alkaloid derived from *Polygonum tinctorium*, *Isatis tinctoria*, and indigo plants [95] and possesses anti-inflammatory, anticancer, and antioxidant effects [96,97,98]. Skin lesion, epidermal thickening, and immune cell infiltration are ameliorated by treatment with TPT in the IMQ-induced psoriasis mouse model [99]. Inflammatory cytokine expression is decreased through the downregulated NF-κB/MAPK pathway following treatment with TPT in an IMQ-induced psoriasis mouse model and TNF-α-exposed human keratinocytes [99]. Moreover, oxidative stress is attenuated through increased levels of antioxidant enzymes and Nrf2/HO-1 activation by treatment with TPT in psoriatic in vivo and in vitro skin models [99];
• 3H-D3T: 3H-1,2-dithiole-3-thione (3H-D3T), a sulfur-containing dithiolethione, is found in cruciferous vegetables and induces antioxidant effects via the Nrf2 signaling pathway [100,101]. Psoriasis symptoms and enhanced epidermal thickness are alleviated by treatment with 3H-D3T in the IMQ-induced psoriasis mouse model [102]. In addition, treatment with 3H-D3T upregulates the Nrf2/HO-1 axis and the activity of SOD and GPx, thereby reducing IL-17-mediated inflammation and mitochondrial oxidative stress in human keratinocytes [102];
• Galangin: Galangin (GGN) is an active flavonoid extracted from *Alpina officinarum*, *Alpina galangal*, and propolis, and possesses various therapeutic effects, including anti-atopic dermatitis [103,104], antioxidants [105], anticolitic [106], anti-inflammatory [107,108], and anti-cancer [109,110] activities. Psoriatic symptoms are attenuated by treatment with GGN in IMQ-induced psoriasis mouse model [111]. Treatment with GGN reduces inflammation through the downregulation of NF-κB inhibitor α (IκBα) degradation-mediated NF-κB activation in the IMQ-induced psoriasis mouse model [111]. Furthermore, oxidative stress is protected by the involvement of GGN-mediated antioxidant enzymes and the Nrf2/HO-1 signaling pathway in the IMQ-induced psoriasis mouse model [111];
• Rutin: Rutin, a common dietary flavonoid, is found in vegetables and most citrus fruits and possesses various biological activities, including antioxidant [112,113] and anti-inflammatory effects [114]. Epidermal hyperplasia, psoriatic symptoms, and inflammatory cell accumulation in the dermis are alleviated by treatment with rutin in the IMQ-induced psoriasis mouse model [115]. Treatment with rutin suppresses inflammation through the upregulation of antioxidant enzyme activity and Nrf2 expression, coupled with the downregulation of Keap1 expression in the IMQ-induced psoriasis mouse model [115]. Conversely, psoriatic symptoms and oxidative stress are exacerbated through Nrf2 knockout by increasing inflammatory cytokine expression and inhibiting antioxidant enzyme activity in the IMQ-induced psoriasis mouse model [115];
• Erianin: Erianin (ERN), a naturally occurring isoflavone derived from *Dendrobium chrysotoxum*, has shown anti-inflammatory, antioxidant, and anti-proliferative activities [116,117,118,119]. Keratinocyte hyperproliferation is inhibited, while keratinocyte differentiation is increased, by treatment with ERN in TNF-α-stimulated human keratinocytes and the IMQ-induced mouse model [120]. The endogenous antioxidant defense system, such as antioxidant-related enzyme activity and Nrf2 signaling, is enhanced by treatment with ERN in the IMQ-induced psoriasis mouse model [120]. Furthermore, treatment with ERN leads to a decrease in the expression of inflammatory cytokines through the suppression of NF-κB, COX-2, and inducible nitric oxide synthase signaling in the IMQ-induced psoriasis mouse model [120];
• Tranexamic acid: Tranexamic acid (TNA) has been used for its antifibrinolytic and antimelanogenic properties [121,122]. In addition, psoriasis symptoms are alleviated by treatment with TNA in the IMQ-induced psoriasis mouse model [123]. Keratinocyte proliferation is decreased through downregulated Nrf2 expression by treatment with TNA in IL-17-stimulated human keratinocytes and the IMQ-induced psoriasis mouse model [123];
• Perillyl alcohol: Perillyl alcohol has been known to inhibit the enzyme farnesyltransferase, thereby inducing anti-inflammatory and antioxidant effects [124]. Moreover, treatment with perillyl alcohol attenuates epidermal hyperplasia and the expression of inflammatory cytokines and proteins, including COX-2, NF-κB, signal transducer and activator of transcription 3 (STAT3), while maintaining GSH, SOD, and Nrf2 levels in keratinocytes in both in vitro and IMQ-induced psoriatic mouse models [124].

Of note, although various therapeutic compounds have been developed and clinically approved, the development of biologic therapies for psoriasis to expand the therapeutic targets remains in progress.

**Table 2:** Mechanisms of bioactive compounds in psoriasis and diverse effects.

Bioactive Compound	Related Mechanism for Alleviation of Psoriasis	Effects	Models	Refs.
Gallic acid	Attenuated K16 and K17	Antioxidant and anti-inflammatory	Human keratinocytes and psoriasis mouse model	[74]
MMF	Induction of Nrf2 expression and enhanced AQP3 expression	Anti-psoriatic	Murine primary keratinocytes	[75]
DMF	Increased p53 expression and decreased Bcl-2 expression, enhanced Nrf2, HO-1, and AKR1C3 expression	Anti-inflammatory, anti-proliferative, and immune-modulatory effects	Human keratinocytes and psoriasis mouse model	[76,77,78,79,80]
Astilbin	Nuclear translocation of Nrf2	Anti-inflammatory, antioxidant, and anti-tumor	Human keratinocytes	[81,82,83,84]
Tussilagonone	Enhanced Nrf2	Anti-inflammatory	Human keratinocytes and psoriasis mouse model	[86]
Ambroxol	Attenuated Ki67 expression	Anti-inflammatory, antioxidant, anti-fungal, anti-bacterial, anti-viral, and anti-fibrotic	Psoriasis mouse model	[87,88,89]
Mn-HyCO/Ru-HyCO	Enhanced Nrf2/HO-1 pathway	Anti-inflammatory	Psoriasis mouse model	[90]
SFN	Nrf2 activation	Antioxidant, anti-cancer, and anti-inflammatory	Psoriasis mouse model and cytokine-stimulated human keratinocytes	[91,92,93,94]
TPT	Downregulated NF-κB/MAPK pathway and Nrf2/HO-1 activation	Anti-inflammatory, anti-cancer, and antioxidant	Psoriatic in vivo and in vitro skin models	[96,97,98,99]
3H-D3T	Upregulated Nrf2/HO-1 axis and activity of SOD and GPx	Antioxidant and anti-inflammatory	Human keratinocytes	[100,101,102]
GGN	Downregulation of IκBα, degradation-mediated NF-κB activation, Nrf2/HO-1 signaling pathway	Anti-atopic dermatitis, antioxidant, anti-colitic, anti-inflammatory, and anti-cancer	Psoriasis mouse model	[103,104,105,106,107,108,109,110,111]
Rutin	Upregulation of Nrf2 expression and downregulation of Keap1 expression	Antioxidant and anti-inflammatory	Psoriasis mouse model	[112,113,114,115]
ERN	Enhanced Nrf2 signaling, suppression of NF-κB, COX-2, and inducible nitric oxide synthase signaling	Anti-inflammatory, antioxidant, and anti-proliferation	Psoriasis mouse model and TNF-α-stimulated human keratinocytes	[116,117,118,119,120]
TNA	Downregulated Nrf2 expression	Anti-fibrinolysis, anti-melanogenesis, and anti-psoriatic	Psoriasis mouse model and IL-17-stimulated human keratinocytes	[121,122,123]
Perillyl alcohol	Maintenance of GSH, SOD, and Nrf2 levels	Anti-inflammatory and antioxidant	Human keratinocytes and psoriasis mouse model	[124]

Abbreviations: K16, keratin16; K17, keratin17; AQP3, aquaporin 3; MMF, monomethyl fumarate; Nrf2, nuclear factor erythroid 2-related factor 2; DMF, Dimethyl fumarate; Bcl-2, B-cell lymphoma 2; HO-1, heme oxygenase-1; AKR1C3, aldo-keto reductase family 1 member C3; Mn-HyCO/Ru-HyCO, manganese-hybrid carbon monoxide/ruthenium-hybrid carbon monoxide; SFN, sulforaphane; TPT, tryptanthrin; NF-κB, nuclear factor kappa B; MAPK, mitogen-activated protein kinase; 3H-D3T, 3H-1,2-dithiole-3-thione; IκBα, NF-κB inhibitor α; SOD, superoxide dismutase; GPx, glutathione peroxidase; GGN, galangin; Keap1, Kelch-like ECH-associated protein 1; ERN, Erianin; COX-2, cyclooxygenase-2; TNF-α, tumor necrosis factor-α; TNA, tranexamic acid; IL-17, interleukin 17; GSH, glutathione.

#### 4.1.2. Various Delivery Platforms in Psoriasis Therapy

**Figure 1:**
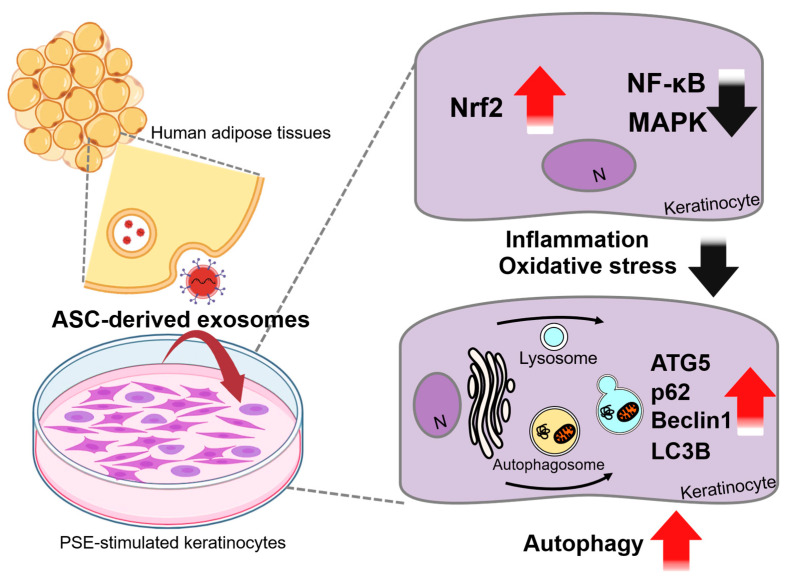
Schematic cellular mechanism of psoriasis treatment with ASC-derived exosomes. PSE-derived keratinocytes present high levels of inflammation and oxidative stress. Treatment with ASC-derived exosomes reduces inflammation and oxidative stress by increasing Nrf2 expression and decreasing NF-κB and MAPK levels. Also, ASC-derived exosomes increase autophagy by upregulation of ATG5, p62, Beclin1 and LC3B. The black bold arrows denote downregulated protein expression or cellular functions and the red bold arrows indicate upregulated protein expression or cellular functions. PSE: psoriasis-derived exosome; ASC: adipose-derived stem cell; Nrf2: nuclear factor erythroid 2-related factor 2; NF-κB: nuclear factor kappa B; MAPK: mitogen-activated protein kinase; N: nucleus; ATG5: autophagy-related gene 5; p62: sequestosome-1; LC3B: microtubule-associated protein 1 light chain 3B.

Drug delivery platforms are required to enhance drug efficacy and sustainability. Recently, several delivery platforms have been studied in psoriatic tissues. For instance, exosomes are 50–150 nm extracellular vesicles that contain nucleic acids, lipids, and proteins and facilitate cell-to-cell communication and membrane transport [125]. The molecular composition of exosomes varies depending on physiological or pathological conditions, cell origin, and tissue type [126]. Thus, patient serum with psoriasis-derived exosome (PSE) induces psoriatic inflammation in human keratinocytes and its inflammation is attenuated through inhibition of NF-κB and MAPK following stimulation with adipose-derived stem cell (ASC)-derived exosomes [127]. PSE-induced oxidative stress is decreased through the upregulated expression of Nrf2 and the inhibition of NF-κB and MAPK by stimulation with ASC-derived exosomes [127]. Moreover, dysregulated autophagy is closely related to the psoriatic process [128,129]. Stimulation with ASC-derived exosomes enhances the autophagy process through the upregulated expression of autophagy protein 5 (ATG5), p62, Beclin1, and LC3B, thereby restoring autophagy regulation in PSE-induced psoriatic keratinocytes [127]. To help to understand exosomal approaches, we summarize the molecular mechanism of ASC-derived exosomes in psoriasis (Figure 1).

**Figure 2:**
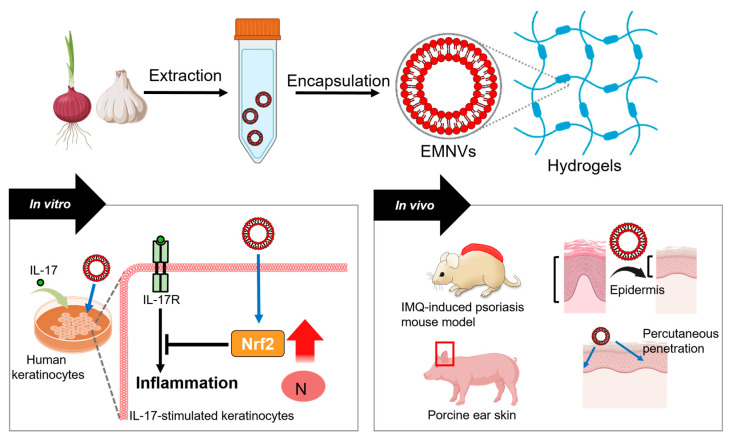
Schematic representation of the effects of hydrogel-based EMNVs from shallot and garlic on IL-17-stimulated human psoriatic keratinocytes and IMQ-induced murine model and porcine ear skin. Treatment with EMNVs reduces inflammation by increasing Nrf2 expression levels. Moreover, treatment with EMNVs attenuates epidermis thickness and elevates percutaneous penetration. The red bold arrows indicate upregulated protein expression of Nrf2. EMNVs: exosome-mimic nanovesicles; IL-17: interleukin 17; IL-17R: receptor of interleukin 17; Nrf2: nuclear factor erythroid 2-related factor 2; N: nucleus; IMQ: imiquimod.

Recently, exosome-mimic nanovesicles (EMNVs) from plants have been developed to enhance bioavailability and drug solubility. EMNVs from shallot and garlic are encapsulated in hydrogels and applied in an IMQ-induced model [130]. Shallot and garlic EMNV hydrogel platforms reduce IL-17-mediated psoriatic inflammation through enhanced Nrf2 expression; they also decrease epidermal thickness and improve percutaneous penetration in the IMQ-induced model and porcine ear skin, respectively [130]. Hydrogel-based NVs from plant compounds have been proposed as effective and cost-available approaches for targeting psoriatic lesions [130]. The application of hydrogel-based NVs is illustrated in Figure 2.

More recently, the hydrogel microneedle technique has been developed to provide low toxicity, controlled release, high permeability, as well as specific targeting [131]. A delivery strategy using a berberine-based nano microneedle has been proposed to treat psoriasis, although direct experimental evidence of the relationship between berberine treatment and Nrf2 was not mentioned in this context [131,132,133]. On the other hand, the Chinese medicinal compound berberine has demonstrated effective properties through the activation of the Nrf2 signaling pathway in several diseases, such as neuronal and hepatic injuries [134,135]. Above all, the development of delivery platforms for skin diseases, including psoriasis and related disorders, has emerged as a therapeutic approach with substantial potential.

### 4.2. Vitiligo

Vitiligo, a common, chronic, and acquired autoimmune skin disease with an estimated prevalence of 1% in the general population [136], is characterized by well-demarcated depigmented macules or patches on the affected skin and mucous membranes [137]. Skin lesions are distributed over the entire body surface; however, they generally occur around the mouth and eyes, as well as on the fingers and dorsum of the feet. Moreover, the hair growing on affected skin areas may turn white [138]. In this section, we summarize the mechanisms associated with vitiligo and the therapeutic approaches to treat vitiligo.

#### 4.2.1. Vitiligo-Associated Mechanisms

The destruction of melanocytes in locally affected skin has been confirmed to be the ultimate cause leading to vitiligo [139]. When oxidative stress including UVB irradiation occurs, excessive oxygen free radicals disrupt cellular proliferation and differentiation and induce immune responses against melanocytes, resulting in irreversible damage, such as apoptosis and the non-apoptotic cell death pathway ferroptosis, in melanin-producing cells [140,141]. Melanocyte function is critically regulated by the Nrf2/HO-1 signaling pathway in the presence of oxidative stress; however, abnormal localization of Nrf2 occurs in vitiligo melanocytes [142,143], suggesting that Nrf2-associated downstream signaling is involved in the pathogenesis of vitiligo. Protecting melanocytes is essential for maintaining skin and hair pigmentation, and we introduce several compounds for the protection of melanin content.

Downregulated AQP3 has been observed in vitiligo keratinocytes [144]. Knockdown of AQP3 and oxidative stress reduces the expression of Nrf2/NQO1 in human epidermal keratinocytes [144]. Media from AQP3-depleted keratinocytes induces the reduction of melanocytes through the enhanced ROS level [144], suggesting crosstalk between keratinocytes and melanocytes should be considered to treat vitiligo. Recently, CXC motif chemokine receptor 3 isoform B (CXCR3B) has been associated with apoptosis of melanocytes [145]. CXC-ligand 10 (CXCL10) is increased in fibroblasts of vitiligo skin and CXCR3B-positive melanocytes are enhanced in active vitiligo skin compared to healthy skin [145]. Enhanced CXCR3B induces apoptosis of melanocytes by impairing Nrf2 regulation [145], suggesting inhibition of CXCR3B could be a therapeutic strategy of vitiligo skin tissue. Additionally, signal transmission between melanocytes and other skin environment is a crucial factor in pathogenesis of vitiligo. Guo H. et al. showed that granulin-sortilin 1 ligand-receptor (GS1LR) is closely related to oxidative stress regulation in the skin environment and reduced expression of GS1LR is observed in vitiligo lesions [146]. Progranulin, encoded by *GRN*, enhances melanocytes resistance to oxidative stress through the involvement of Nrf2 and HO-1 [146]. Although the mechanism of vitiligo has been gradually identified in recent years, we summarize several therapeutic compounds that regulate Nrf2-associated redox state against vitiligo in melanocytes to expand the field of vitiligo treatment (Table 3).

#### 4.2.2. Therapeutic Compounds in Melanocyte Regulation

• Simvastatin: Simvastatin is considered an effective antioxidant compound and enhances the Nrf2/ARE signaling pathway [147]. Treatment with simvastatin induces anti-oxidative effects across various oxidative stress-related diseases [148]. In addition, simvastatin attenuates H_2_O_2_-induced melanocyte apoptosis through the activation of Nrf2 and subsequent enhancement of NQO1/HO-1, as well as the involvement of activated MAPK and p62 signaling pathways [148];
• Vitamin D: 25-hydroxyvitamin D (vitD) has been known to possess therapeutic potential in various conditions, such as osteoporosis, cancer, and autoimmune diseases [149]. Notably, insufficiency of vitD is closely related to progression of vitiligo [150]. VitD modulates Wnt/β-catenin signaling in oxidative stress-stimulated melanocytes and significantly attenuates ROS level and apoptosis in H_2_O_2_-stimulated melanocytes [150]. Moreover, treatment with vitD activates the Nrf2/ARE signaling pathway against oxidative stress [150], suggesting that treatment with vitD protects the melanocytes from ROS-induced apoptosis through the Wnt/β-catenin/Nrf2/ARE signaling pathway and thus could be a potential treatment strategy against vitiligo;
• *Lycium barbarum* polysaccharide: Goji berries are the mature, dried fruit of *Lycium barbarum* L., a deciduous shrub in the *Solanaceae* family [151]. *L. barbarum* polysaccharide (LBPS) is a major pharmacological constituent known for its antioxidant and anti-photoaging activities [152,153,154]. H_2_O_2_-mediated apoptosis is attenuated by treatment with LBPS in human melanocytes [151]. Furthermore, melanin content is enhanced through the upregulation of tyrosinase activity in H_2_O_2_-stimulated human melanocytes [151]. Mechanistically, treatment with LBPS enhances the autophagy process by upregulating the expression of Beclin1 and several autophagy-related genes (ATGs 5, 7, 12, and 16L1), increasing the number of autophagic vacuoles, and activating Nrf2/p62 signaling, thereby promoting melanocyte proliferation in H_2_O_2_-stimulated human melanocytes [151];
• Roflumilast: Roflumilast, a phosphodiesterase 4 (PDE4) inhibitor, has been applied to various skin diseases, such as atopic dermatitis [155] and oxidative stress-associated neuronal damage [156,157]. Treatment of roflumilast attenuates oxidative stress-induced apoptosis in melanocytes by enhancing Nrf2 signaling [158]. Moreover, the combined treatment of roflumilast and forskolin boosts intracellular cyclic adenosine monophosphate (cAMP) levels and protects the apoptosis of melanocytes [158], suggesting modulation of cAMP levels could be a potential strategy against vitiligo;
• Paeoniflorin: The anti-photoaging activity of paeoniflorin has been addressed in Section 3. In the context of vitiligo treatment, H_2_O_2_-induced apoptosis is attenuated by treatment with paeoniflorin in both melanocytes and vitiligo melanocytes [159]. In addition, oxidative stress is reduced through increased SOD and CAT activity and the activation of the JNK/Nrf2/HO-1 pathway following treatment with paeoniflorin in H_2_O_2_-stimulated human melanocytes [159];
• Baicalein: Baicalein is a flavone derived from the roots of *Scutellaria baicalensis* [160] and possesses various biological activities, such as anti-oxidative, antitumor, and anti-inflammatory activities [161,162,163]. H_2_O_2_-induced apoptosis and mitochondria dysfunction are mitigated by treatment with baicalein through the activation of Nrf2 signaling in human vitiligo melanocytes [164];
• Vitexin: Vitexin is a C-glycosylated flavonoid compound derived from medicinal plants such as hawthorn, pearl millet, and *Herba spirodelae* [165,166] and has been reported to possess various biological activities, including antioxidant and anti-inflammatory activities [167,168]. H_2_O_2_-induced oxidative stress is attenuated by vitexin treatment through the activation of Nrf2/HO-1 signaling, thereby reducing apoptosis by upregulating p53 and Bcl-2 levels while downregulating Bax and cleaved caspase-3 levels in human melanocytes [169]. Additionally, treatment with vitexin decreases inflammation by upregulating the Nrf2/ARE axis in H_2_O_2_-stimulated human melanocytes [169];
• Apigenin: Apigenin is a flavonoid found in the buds and flowers of *Hypericum perforatum* [170] and possesses anti-oxidative and anti-inflammation properties [171]. H_2_O_2_-induced oxidative stress is attenuated by treatment with apigenin through the enhancement of the Nrf2/HO-1/NQO1 signaling pathway in human vitiligo melanocytes [172].

**Table 3:** Mechanisms of bioactive compounds in vitiligo and diverse effects.

Bioactive Compound	Related Mechanism for Alleviation of Vitiligo	Effects	Model	Refs.
Simvastatin	Activation of Nrf2/NQO1/HO-1 and activated MAPK and p62	Antioxidant	H_2_O_2_-treated melanocytes	[147,148]
Vitamin D	Activation of Wnt/β-catenin/Nrf2/ARE signaling	Anti-osteoporosis, anti-cancer, and anti-autoimmunity	Oxidative stress-stimulated melanocytes	[150]
*Lycium barbarum* polysaccharide	Upregulated tyrosinase activity, expression of Beclin1, autophagy-related genes, the number of autophagy vacuoles, and Nrf2/p62 signaling	Antioxidant and anti-photoaging	H_2_O_2_-stimulated human melanocytes	[151,152,153,154]
Roflumilast	Enhanced Nrf2 signaling	Anti-atopic dermatitis and treatment of oxidative stress-associated neuronal damage	Oxidative stress-stimulated melanocytes	[155,156,157,158]
Paeoniflorin	Increased antioxidant enzymes activity and activated JNK/Nrf2/HO-1 pathway	Anti-inflammatory, anti-apoptotic, and antioxidant	H_2_O_2_-stimulated human melanocytes	[46,47,48,159]
Baicalein	Nrf2 signaling	Anti-oxidative, antitumor, and anti-inflammatory activities	H_2_O_2_-exposed human vitiligo melanocytes	[161,162,163,164]
Vitexin	Activated Nrf2/ARE/HO-1 signaling, upregulated p53 and Bcl-2 levels, and downregulated Bax and cleaved caspase-3 levels	Antioxidant and anti-inflammatory activities	H_2_O_2_-stimulated human melanocytes	[167,168,169]
Apigenin	Enhanced Nrf2/HO-1/NQO1 signaling pathway	Anti-oxidative and anti-inflammation properties	H_2_O_2_-stimulated human vitiligo melanocytes	[171,172]

Abbreviations: Nrf2, nuclear factor erythroid 2-related factor 2; NQO1, NAD(P)H:quinone oxidoreductase 1; HO-1, heme oxygenase 1; MAPK, mitogen-activated protein kinase; p62, sequestosome 1; H_2_O_2_, hydrogen peroxide; Wnt, wingless and Int-1; ARE, antioxidant response element; JNK, c-Jun N-terminal kinase; Bcl-2, B-cell lymphoma 2; Bax, Bcl-2-associated X protein.

#### 4.2.3. Non-Apoptotic Cell Death Ferroptosis on Melanocytes in Vitiligo

Ferroptosis, characterized by iron-dependent lipid peroxidation, is closely associated with the loss of melanocytes and subsequent depigmentation [173,174]. Recently, ferroptotic-specific gene markers, including Nrf2, have been identified in vitiligo [174,175]. Several nature-derived compounds targeting this pathway have been proposed as potential treatments for ferroptosis-associated vitiligo. For instance, a polyphenolic compound derived from the plant *Curcuma longa* Linn [176], curcumin, possesses anti-cancer, anti-inflammatory, and antioxidant properties [177]. As a treatment strategy for vitiligo, the effect of curcumin is evaluated by the erastin-induced ferroptosis in an in vitro model in melanocytes [178]. Treatment with curcumin induces the nuclear translocation of Nrf2 and the expression of HO-1, protecting melanocytes against erastin-mediated ferroptotic cell death [178]. Moreover, tanshinone IIA, extracted from the Chinese medicinal herb *Salvia miltiorrhiza* Bunge [179], attenuates cytotoxic activity of CD8(+) T cells, thereby suppressing the autoimmune condition associated with vitiligo [180]. More recently, treatment with tanshinone IIA inhibits H_2_O_2_-mediated ferroptosis through the activation of the Nrf2 signaling pathway in melanocytes [181]. Although the development of therapeutic candidates for vitiligo remains necessary, targeting ferroptosis-associated melanocyte death could provide a mechanistic approach to prevent melanocyte loss and represents a potential therapeutic strategy against vitiligo.

### 4.3. Autoimmune-Associated Skin Fibrosis

Skin fibrosis occurs in various conditions, including systemic sclerosis, hypertrophic scars, and keloid scars. Among these, systemic sclerosis is a chronic rheumatic disease characterized by immune dysregulation, leading to widespread fibrosis involving both the skin and internal organs [182]. The pathogenesis of systemic sclerosis has been linked to dysregulated oxidative stress regulation [183]. To recover the clinical manifestation of fibrosis in systemic sclerosis, the modulation of Nrf2 has been identified as a critical therapeutic target. These mechanisms and associated compounds are summarized in Table 4.

Reduced Nrf2 expression has been revealed in patients with systemic sclerosis [183]. Mechanically, Nrf2 blocks transforming growth factor-β (TGF-β) signaling, which is responsible for inducing myofibroblast proliferation [183]. Thus, the Nrf2 agonist 2-trifluoromethyl-2′-methoxychalcone inhibits TGF-β-induced dermal fibrosis [183]. Xanthohumol has been used for treatment of fibrotic diseases as well as for various pharmacological effects, including antioxidant, anti-inflammatory, and anti-proliferative properties [184,185], and applied to modulate skin fibrosis in systemic sclerosis [186]. Xiao Y. et al. showed that systemic sclerosis fibroblasts increase collagen production in vitro [186]. Treatment with xanthohumol enhances ROS production and Nrf2 expression, thereby inhibiting collagen production through the inhibition of the TGF-β/SMAD3 signaling pathway in systemic sclerosis-derived fibroblasts [186]. Moreover, enhanced expression of serine/threonine kinase Pim-1 proto-oncogene (PIM1) is revealed in skin fibrosis-associated tissues [187]. PIM1 is also associated with liver oxidative stress through the modulation of the Nrf2/NQO1/HO-1 pathway [188]. Wang J. et al. performed a high-throughput screening assay and found bruceine D as a PIM1-binding small molecule [187]. Bleomycin (BLM) treatment is a well-established method to induce fibrosis and is widely used as an experimental fibrosis model [189]. Treatment with bruceine D induces ferroptosis in hypertrophic scar fibroblasts by inhibiting the Pim-1 proto-oncogene (PIM1)/Nrf2 signaling pathway and effectively suppresses fibrosis in the BLM-induced fibrotic model [187], suggesting that bruceine D possesses therapeutic potential for abnormal PIM1 expression-associated fibrotic diseases.

Radiation therapy has been considered an effective strategy against cancer. However, its side effects occur systemically. For instance, radiation-induced dermatitis progresses to fibrosis, a process primarily driven by oxidative stress. To recover radiation-mediated skin damage, the therapeutic candidate ON101, a formulation containing extracts from *Plectranthus amboinicus* and *Centella asiatica*, has been investigated [190]. Topical application of ON101 cream reverses oxidative damage in radiation-induced murine dermatitis through the inhibition of Keap1 and enhancement of the Nrf2 pathway [190]. Moreover, treatment of ON101 induces reduced ROS levels and inflammatory cytokines through the modulation of Keap1/Nrf2 signaling in both HaCaT keratinocytes and dermal fibroblasts [190], suggesting that ON101 could be a therapeutic candidate against skin fibrosis.

**Table 4:** Mechanisms of bioactive compounds in skin fibrosis and diverse effects.

Bioactive Compound	Related Mechanism for Alleviation of Skin Fibrosis	Effects	Models	Refs.
Xanthohumol	Upregulation of Nrf2 expression, inhibition of TGF-β/SMAD3 signaling pathway	Antioxidant, anti-inflammatory, and anti-proliferative	Systemic sclerosis-derived fibroblasts	[186]
Bruceine D	Inhibition of PIM1/Nrf2 signaling	Ferroptosis inducer	Fibrotic model	[187]
ON101	Inhibition of Keap1 and enhancement of Nrf2 pathway	Promotion of wound healing	Human keratinocytes and dermal fibroblast	[190]

Abbreviations: Nrf2, nuclear factor erythroid 2-related factor 2; TGF-β, transforming growth factor-β; SMAD3, mothers against decapentaplegic homolog 3; PIM1, Pim-1 proto-oncogene, serine/threonine kinase; Keap1, Kelch-like ECH-associated protein 1.

## 5. Conclusions

**Figure 3:**
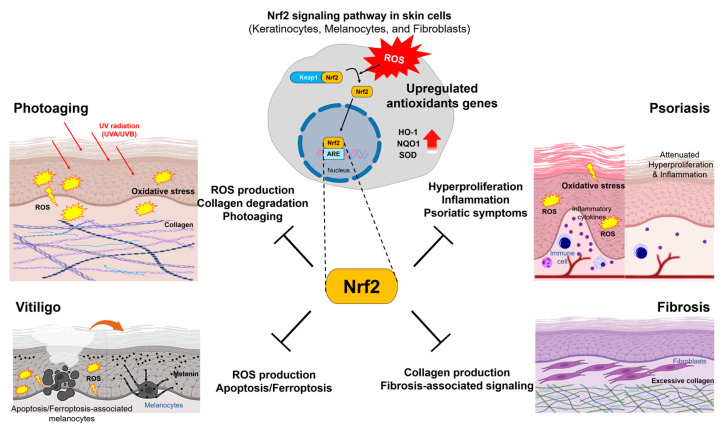
Schematic cellular mechanism of Nrf2 in skin cells. Oxidative stress-mediated Nrf2 activation upregulates antioxidant genes, and subsequently inhibits skin photoaging, psoriasis, vitiligo, and fibrosis. The red bold arrows indicate upregulated protein expression. ROS: reactive oxygen species; Keap1: Kelch-like ECH-associated protein 1; Nrf2: nuclear factor erythroid 2-related factor 2; ARE: antioxidant response elements, HO-1: heme oxygenase-1; NQO1: NAD(P)H:quinone oxidoreductase 1; SOD: superoxide dismutase.

Oxidative stress within skin tissue is mainly associated with aging and various cutaneous pathologies. Modulation of stress signals is crucial for treating various skin damage and disease. Dysregulation of the Nrf2 signaling pathway has been implicated in a wide range of cutaneous pathologies, including photoaging, psoriasis, vitiligo, and fibrosis. In photoaging, Nrf2 protects against UV-induced oxidative stress and DNA damage; however, it may also influence extracellular matrix remodeling. In psoriasis, impaired Nrf2 function contributes to keratinocyte hyperproliferation, inflammatory cytokine production such as IL-17 [102,130], and compromised skin barrier function. Moreover, in vitiligo, oxidative stress-induced melanocyte loss is closely linked to a defective Nrf2-mediated antioxidant defense system. In skin fibrosis, aberrant Nrf2 activity either suppresses or exacerbates fibroblast activation, depending on the specific cellular context and disease stage. Collectively, these findings highlight Nrf2 as a pivotal component connecting oxidative stress to immune dysregulation, aberrant cell proliferation, and extracellular matrix changes in diverse skin disorders and the integrated role of Nrf2 across these diverse skin disorders is illustrated in Figure 3. Thus, an understanding of oxidative stress-regulating component Nrf2 will be prominent in the process of development of modulatory drugs and the application of diverse delivery approaches.

Here, we provide several perspectives on Nrf2 modulation in skin disorders, ranging from aging to autoimmune-related diseases. For the specific modulation of Nrf2, the dual role of Nrf2 (protective vs. pathogenic) in fibrotic skin diseases and chronic inflammatory conditions necessitates a precise delineation of its cell-type- and stage-specific effects. For the therapeutic targeting of Nrf2, activators of Nrf2 (e.g., sulforaphane) possess therapeutic potential [94]; however, rigorous evaluation of topical versus systemic delivery, long-term safety, and disease-specific efficacy is required. Evaluation of crosstalk between Nrf2-associated immune and metabolic pathways will be required. Moreover, the combination of Nrf2 modulators with other therapeutic modulators (e.g., JAK inhibitors [191], anti-cytokine biologics) possesses the potential for synergistic efficacy in multifactorial skin disorders; however, vigorous mechanistic studies and massive clinical trials are required to establish safety and therapeutic benefits. Moreover, development of clinical case studies in complex cutaneous effects is required because a one-sided skin therapeutic approach may paradoxically trigger or aggravate other cutaneous diseases [192]. Future research should focus on spatiotemporal regulation of Nrf2, novel delivery strategies for Nrf2 modulators, and integrative/complex omics approaches [193,194] which would be key procedures to translate basic insights into clinical interventions for oxidative stress-driven skin damage and diseases.

## Data Availability

No new data were created or analyzed in this study. Data sharing is not applicable to this article.
